# Evaporation and drying kinetics of water-NaCl droplets *via* acoustic levitation

**DOI:** 10.1039/c9ra09395h

**Published:** 2020-01-08

**Authors:** Yutaro Maruyama, Koji Hasegawa

**Affiliations:** Graduate School of Engineering, Kogakuin University Tokyo 163-8677 Japan; Department of Mechanical Engineering, Kogakuin University Tokyo 163-8677 Japan kojihasegwa@cc.kogakuin.ac.jp

## Abstract

The acoustic levitation method (ALM) is expected to be applied as a container-less processing technology in the material science, analytical chemistry, biomedical technology, and food science domains because this method can be used to levitate any sample in mid-air and prevent nucleation and contamination due to the container wall. However, this approach can lead to nonlinear behavior, such as acoustic streaming, which promotes the evaporation of a levitated droplet. This study aims to understand the evaporation and precipitation kinetics of an acoustically levitated multicomponent droplet. An experimental investigation of the evaporation process of a salt solution droplet was performed, and the experimental results were compared with those of the *d*^2^-law. The droplet was noted to evaporate in two stages owing to the precipitation of the salt. Because of the vapor pressure depression, the experimental data did not agree with the classical prediction obtained using the *d*^2^-law. However, the experimental results were in partial agreement with those of the *d*^2^-law when the vapor pressure depression was considered by using the concentration estimate at each time, as obtained from the experimental results. In addition, it was observed that the time when the salt completely precipitated could be estimated by using the extended theory. These findings provide physical and practical insights into the droplet evaporation mid-air for potential lab-in-a-drop applications.

## Abstract 2

The droplet levitation dynamics associated with the evaporation and precipitation facilitate a more universal understanding for potential lab-in-a-drop applications.

## Graphical abstract

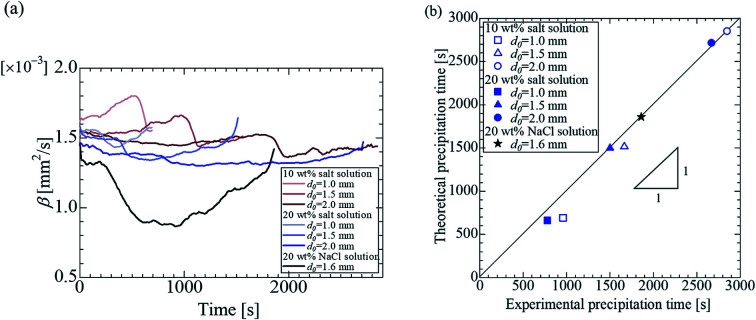

## Introduction

Evaporation of droplets is a fundamental phenomenon in nature. The dynamics of evaporation for sessile droplets on a solid substrate have been extensively studied over the past decades.^1–5^ While it is useful to investigate the evaporation behavior of sessile droplets, complex wetting dynamics by the interaction between the droplet and solid substrate should also be taken into account to understand the nature of the evaporation. Ideally, it is desirable to investigate the evaporation dynamics in mid-air without the wall effect. ALM is a non-contact technology that can be used to suspend a droplet near a sound pressure node of a standing wave through the action of acoustic radiation force.^6,7^ Because this technology can avoid the adsorption, contamination, and heterogeneous nucleation caused by the vessel wall surface, it is expected to be used to realize non-contact manipulation in fields such as material creation,^8,9^ chemistry,^10,11^ and biomedicine.^12,13^ Yu *et al.* successfully synthesized single-atom Pt materials in solution and supported Pt nanoclusters on microporous L_2_O_3_ by using a one-step ALM without any pretreatment/modification of raw oxide.^14^ Zang *et al.* used ALM to generate bubbles for industries pertaining to inorganic salts, food, cosmetics, and materials.^15,16^ Furthermore, these authors also developed a microreactor that coats solid potassium permanganate on the surface of levitated water droplets.^17,18^ Xie *et al.* demonstrated that small organisms such as insects and small fish can levitate in a living state.^19^ Sundvik *et al.* studied the effects of hatching and growth processes on levitated zebrafish embryos, which are frequently used as a biological model of small animals in experiments. The results indicated that the ALM did not exert any adverse effect on the growth and main organs of the zebrafish, and the method has thus been employed for transporting and observing organisms in a container-less state while avoiding contamination.^20^ These findings indicate that the use of ALM to perform non-contact manipulation in the fields of material generation, chemistry, and biomedicine has been investigated. Furthermore, in recent years, non-contact manipulation by using an ultrasonic phased array has also been investigated, thereby expanding the application of the ALM for non-contact manipulation.^21,22^

However, the use of the ALM leads to the introduction of nonlinear dynamics on a levitated droplet, such as in the form of acoustic streaming^23–28^ and dynamic behavior,^29,30^ owing to the levitation of the sample by a nonlinear acoustic field. These phenomena may affect the evaporation and precipitation of the levitated samples. Yarin *et al.* found in a theoretical study that the flow occurring around levitated droplets affects the mass transport.^31^ Hasegawa *et al.* studied the correlation between the flows inside and outside the levitated binary droplet under the evaporation process.^32^ Kobayashi *et al.* visualized that a correlation exists between the flow structures of the droplet and the vapor concentration with an interferometer.^33^ Bänsch *et al.* studied the evaporation behavior of levitating droplets by using numerical simulations, taking into account the deformation of the droplets owing to the heat and mass transport at the droplet interface, acoustic flow, and acoustic radiation pressure.^34^ Zaitone proposed a theoretical model for the evaporation of spheroidal droplets, considering the effect of the acoustic flow.^35^ Yarin *et al.* developed an evaporation model for multicomponent droplets levitated in an acoustic field.^36^ Niimura *et al.* observed the evaporation process of single-component and multicomponent droplets without precipitation, and based on the results, the existing theory was extended for the evaporation of multicomponent droplets.^37^ In addition, many studies have been conducted to clarify the evaporation kinetics of droplets corresponding to the use of the ALM.^38–42^ Furthermore, an evaporation process involving precipitation was investigated by Combe *et al.*, who estimated the evaporation process of droplets containing salt components and compared the results with those obtained using the theory considering the effect of the solute.^43^

Although many researchers have investigated the evaporation process of the pure and binary droplet, the investigation on the droplet evaporation with precipitation in mid-air remains challenging. This study aimed to understand the evaporation phenomenon and precipitation kinetics of multicomponent droplets *via* acoustic levitation. In addition to the performance of an experiment involving the quantification of the evaporation process by using a high-speed camera and radiation thermometer, the experimental results were compared with those obtained using the classical (*d*^2^-law) and expanded theories. The presented droplet levitation dynamics associated with the evaporation and precipitation may facilitate a more universal understanding for potential lab-in-a-drop applications, such as a microreactor.

## Experimental methodology

**Fig. 1:**
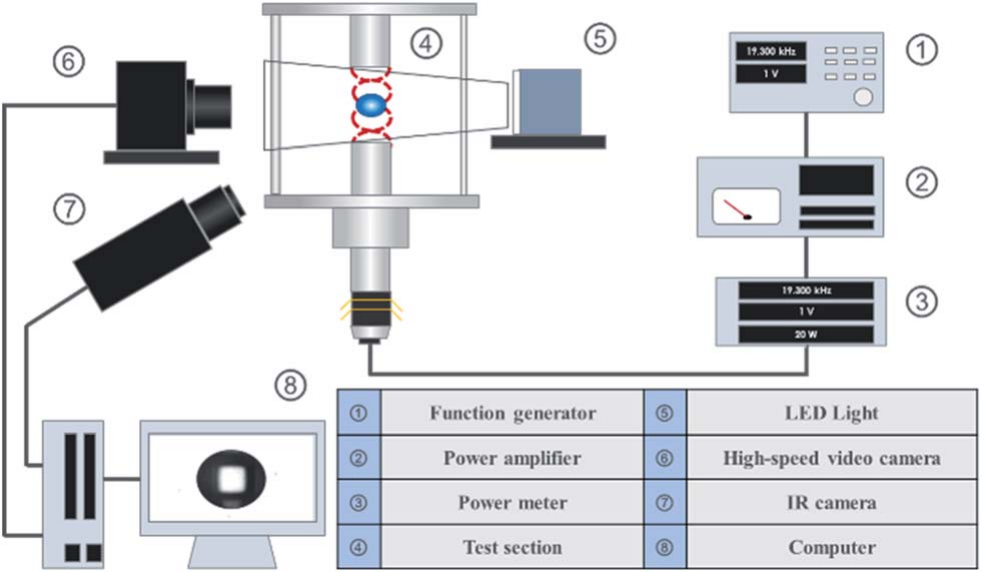
Schematic of experimental setup.

Fig. 1 shows a schematic of the experimental apparatus employed in this study. A sine wave signal, generated by a function generator (Agilent Technologies Japan 33511B), was amplified using an amplifier (NF Corporation 4502) and input to an ultrasonic transducer (NGK Spark Plug Co. D4520PC). The input voltage and amplified power were monitored by using a power meter (Yokogawa Test & Measurement Corporation WT310-DC1). An acoustic standing wave was formed between the under horn connected and the top reflector. When a sample was manually injected using a syringe near a node of the sound pressure field, the droplet could be levitated in mid-air. To visualize the levitated droplets, a high-speed camera (Photron FASTCAM AX50 type HS-TT) with backlight illumination was employed. At the same time, each of the droplets was observed using a radiation thermometer (FLIR Systems A6750sc MWIR). The interface temperature at the time was also measured. Subsequently, the image group obtained by the high-speed camera was processed using a computer. During the image processing, the obtained image group was binarized using the image processing software ImageJ to determine the interface, and the image was processed using the MATLAB Image Processing Toolbox to detect the major and minor diameters of the droplets. The diameters were calculated and converted into a volume equivalent diameter. For the droplet diameter 
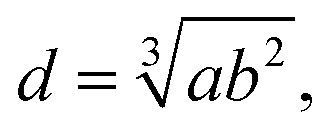
 the volume equivalent diameter when the droplet was assumed to be a spheroid with a minor axis *a* and a major axis *b* was adopted. A probe microphone (Brüel & Kjær Type 4182) was used to measure the sound pressure in the test section.

The sine wave generated by the function transmitter had a frequency of approximately 19.3 kHz, and the distance between the horn and the reflector was 48 mm. The sound pressure of the belly above the suspended droplet, as measured using the probe microphone, was 1.3–1.8 kPa. The wavelength of the test section of the levitating device was 18 mm. The test fluids included a salt solution in which edible salt (Ako Kasei Co., Ltd.) was dissolved in pure water and an NaCl aqueous solution in which NaCl (Hayashi Pure Chemical Ind., Ltd. purity 99.5%) was dissolved. The room temperature was set to 25 ± 2 °C using an air conditioner, and the relative humidity was set to 50 ± 7% using a dehumidifier. The initial droplet diameter was 1.0–2.0 mm. The emissivity for the radiation thermometer was 0.96.^44,45^

## Theory

### Single-component droplets

For non-contact manipulation, it is particularly important to estimate the evaporation of droplets. Therefore, the theoretical and experimental results were compared. The *d*^2^-law,^46^ as an equation for the mass transport of single-component droplets, is widely used in the estimation of the evaporation of single-component droplets, and it can be expressed as follows.1
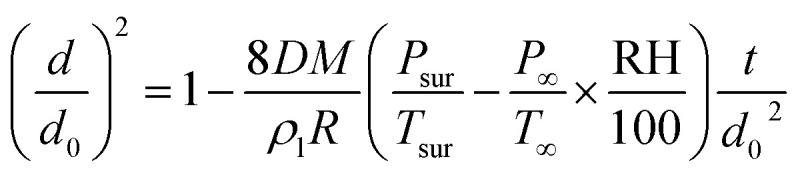
where *D* is the diffusion coefficient, *M* is the molecular weight, *ρ*_l_ is the density, *R* is the gas constant, *P* is the vapor pressure, *T* is the temperature, RH is the relative humidity, *t* is the time, and *d*_0_ is the initial equivalent diameter of the droplet. The subscript sur represents the droplet surface and ∞ represents the ambient gas.

### Multi-component droplet with vapor pressure depression

Based on eqn (1), Combe and Donaldson applied the *d*^2^-law in a form corresponding to the evaporation process of the acoustic levitated droplets involving precipitation.^43^ In the solutions containing non-volatile solutes, the vapor pressure depends on the molar fraction of the solute. Therefore, the *d*^2^-law considering the molar fraction of the solute can be presented as in eqn (2).2
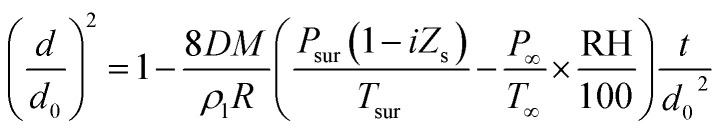
where *Z*_s_ is the solute molar fraction, and *i* is the van't Hoff factor.

## Results and discussion

### General observation

**Fig. 2:**
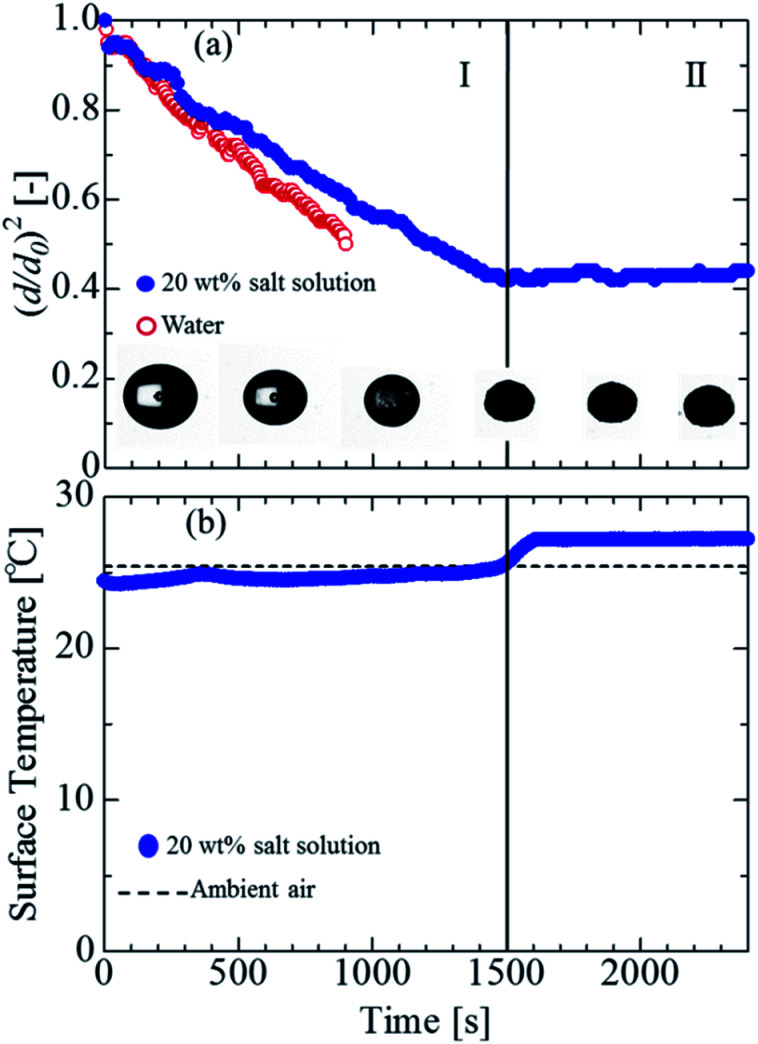
(a) Evaporation and precipitation process of water and salt solution with snapshots of levitated sample. (b) Surface temperature of the salt solution droplet as a function of time. The evaporation process is divided into stages I and II. Stage I represents the evaporation, and stage II represents the full evaporation and precipitation.

The evaporation process and surface temperature were observed using a solution involving salt mixed with pure water. Fig. 2(a) shows the visualization images at each time by using the salt solution (20 wt%). In the salt solution, the snapshot of the droplet was darker due to the precipitation of the salt with time. Subsequently, at approximately 1500 s, it was not possible to confirm the transmission by the backlight. These findings indicate that the salt was completely precipitated at approximately 1500 s.

Fig. 2(a) shows the evaporation process of the salt solution and water. The horizonal axis represents the time, and the vertical axis represents the squared droplet diameter normalized by the squared initial droplet diameter. The initial droplet diameters *d*_0_ of the salt solution and water were 1.5 mm and 1.7 mm, respectively. In stage I, the evaporation process of the water droplets was linear, in which the dimensionless surface area decreased by approximately 50% in 900 s. The evaporation process of the salt solution involved vaporization up to approximately 1500 s, and the dimensionless surface area decreased by approximately 60% at 1500 s. Subsequently, in stage II, the reduction in the dimensionless surface area was nearly eliminated, and the area became constant. This phenomenon occurred because the water component of the salt solution evaporated over time, and it was completely evaporated at approximately 1500 s. Such a two-stage evaporation process was likely occurred because the precipitation salt remained in the solution.

Fig. 2(b) shows the measurement results of the interface temperature of a salt solution droplet. At the time of droplet injection, the temperature was approximately 24 °C, which is 1 °C lower than the outside temperature. Subsequently, although the surface temperature of the salt solution did not exhibit a significant change until approximately 1500 s, it increased to approximately 27 °C from 1500 s to 1605 s and then became constant. The reasons for the increase in temperature between 1500 s and 1605 s and subsequent constancy are believed to be the evaporation of the water component and complete precipitation of the salt. The reason for the surface temperature becoming constant at a temperature 2 °C higher than the room temperature is thought to be due to a deviation from the initial emissivity of 0.96 (calibrated for initial multicomponent droplets), owing to the complete precipitation of the salt. These results indicate that the salt was completely deposited in 1500 s, and the salt could be precipitated in a levitated state.

### Effect of initial concentration

**Fig. 3:**
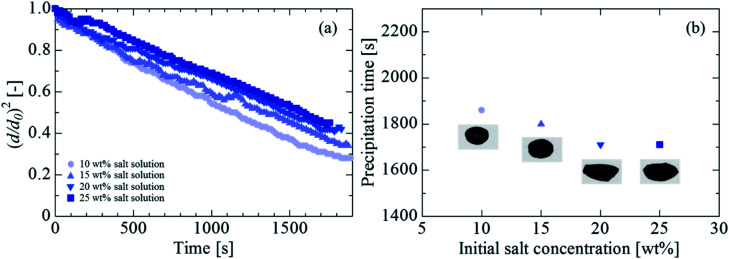
Effect of initial concentration on the droplet evaporation and precipitation. (a) Evaporation process of the salt solution. (b) Precipitation time and final shape of salt.

Fig. 3(a) shows the evaporation process at each initial concentration of 10 wt%, 15 wt%, 20 wt%, and 25 wt% for an initial droplet diameter *d*_0_ of 1.7 mm, 1.7 mm, 1.8 mm, and 1.8 mm, respectively. As the initial concentration increased, the evaporation of the droplet reduced, likely because the evaporation was suppressed by the vapor pressure depression that increased the initial concentration. Fig. 3(b) shows the time taken for the complete salt precipitation at each initial concentration. The horizontal axis in the figure represents the initial salt concentration, and the vertical axis represents the time at which the salt was completely precipitated. The images in the figure are examples of the visualized image at each density. Although the precipitation time was considered to be reduced owing to the decrease in the volume of the water concentration with an increase in the salt concentration (the precipitation time was approximately 1860 s, 1800 s, 1710 s, and 1710 s at 10 wt%, 15 wt%, 20 wt%, and 25 wt%, respectively), no significant change in the precipitation time was observed with a change in the salt concentration. The precipitation time was not reduced likely because the evaporation rate was retarded due to the vapor pressure depression when the salt concentration increased. The visualization images at the time of precipitation indicate that for the salt concentrations of 10 wt% and 15 wt%, the precipitation occurred in a nearly spherical shape, whereas for 20 wt% and 25 wt%, the precipitation shape was closer to an ellipsoid. The shapes are considered to be attributed to the influence of the internal circulation driven by nonlinear sound waves.^24,27^ The applied sound pressures at 10 wt%, 15 wt%, 20 wt%, and 25 wt% were 1.8 kPa, 1.7 kPa, 1.4 kPa, and 1.5 kPa, respectively. The maximum difference was 0.4 kPa. A higher sound pressure corresponded to a higher circulation speed. Therefore, it is considered that concentrations of 10 wt% and 15 wt%, involving a higher internal stirring, led to a spherical precipitation, whereas those of 20 wt% and 25 wt% led to an elliptical precipitation.

### Effect of initial droplet diameter

**Fig. 4:**
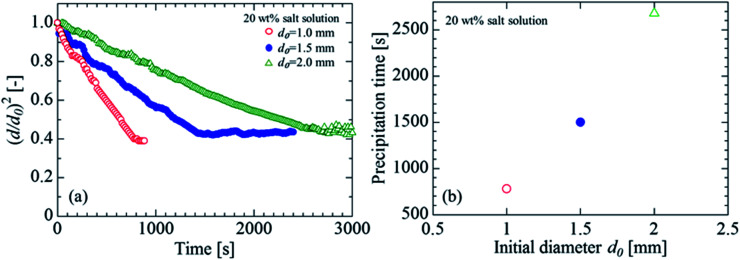
Effect of initial droplet diameter on droplet evaporation and precipitation. (a) Evaporation process of salt solution. (b) Precipitation time.

Fig. 4(a) shows the evaporation process at each initial droplet size for an initial concentration of 20 wt%. The initial droplet diameters *d*_0_ were 1.0 mm, 1.5 mm, and 2.0 mm. The evaporation rate decreased as the initial droplet size increased, likely because the surface area per unit volume decreased as the droplet size increased. Fig. 4(b) shows the effect of the initial droplet size on the salt precipitation. It was confirmed that the precipitation time (when the salt was completely precipitated) was higher for the cases in which the initial droplet diameter was larger.

### Comparison with theory

**Fig. 5:**
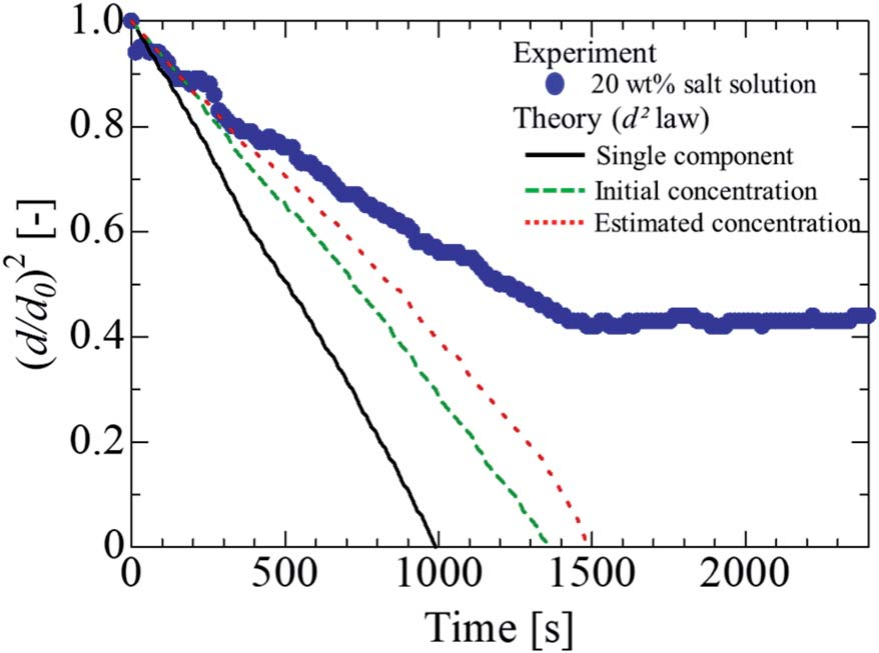
Comparison of theoretical and experimental values.

The evaporation process of the salt solution was compared with the theory represented by eqn (1). For the surface temperature of the droplet, the experimental data in Fig. 2(b) were substituted, and the physical properties of water were used to determine the diffusion coefficient and density of the levitated samples. Fig. 5 presents the comparison between the experimental results and the theoretical prediction. The solid line in the figure indicates the theoretical values obtained using eqn (1). The experimental data were considerably different from the theoretical values, likely because of the effect of the vapor pressure depression due to the dissolution of the non-volatile substances. It is considered that the salt solution was separated because the evaporation rate was lower than that of pure water, owing to the vapor pressure depression. Therefore, it is necessary to expand eqn (1) to take into account the vapor pressure depression.

Consequently, we compared the extended theory represented by eqn (2) with the experimental results, considering the vapor pressure depression. The green dashed line in Fig. 5 shows a comparison between the theoretical and experimental values, considering the vapor pressure drop of the initial concentration. *Z*_s_ was calculated using the initial solute concentration of 20 wt% in the salt solution, and the concentration change with time was not considered. In the calculation, it was assumed that the salt was NaCl, and *i* was 2.^43^ The experimental data and theoretical prediction agreed up to approximately 345 s; however, these values exhibited a discrepancy thereafter. This phenomenon is considered to be because the vapor pressure further decreased due to the increase in the concentration accompanying the evaporation of the salt solution, and the experimentally obtained evaporation rate reduced. For a better prediction of the evaporation kinetics, the concentration change with time was estimated. First, the mass of the water component and salt contained in the saline droplet were calculated using eqn (3) and (4), respectively.3
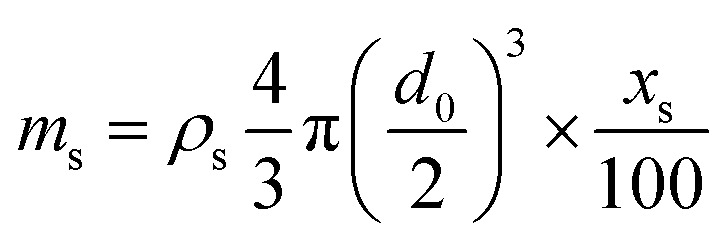
4
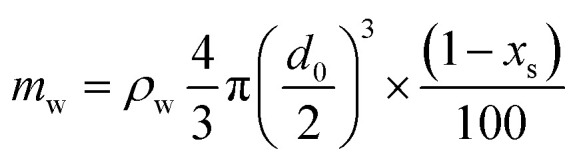
where *ρ* is the density of the NaCl aqueous solution, *d*_0_ is the initial droplet diameter, and *x* is the initial concentration of the solute. The subscripts s and w represent the salt and water components, respectively. The experimental results indicated that the salt solution was completely precipitated in 1500 s; consequently, the mass of each component at each time was calculated such that the mass of the water component evaporated in 1500 s. Eqn (5) shows the formula to calculate the mass fraction used.5
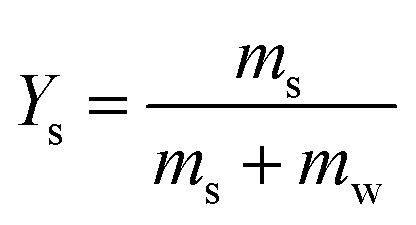


**Fig. 6:**
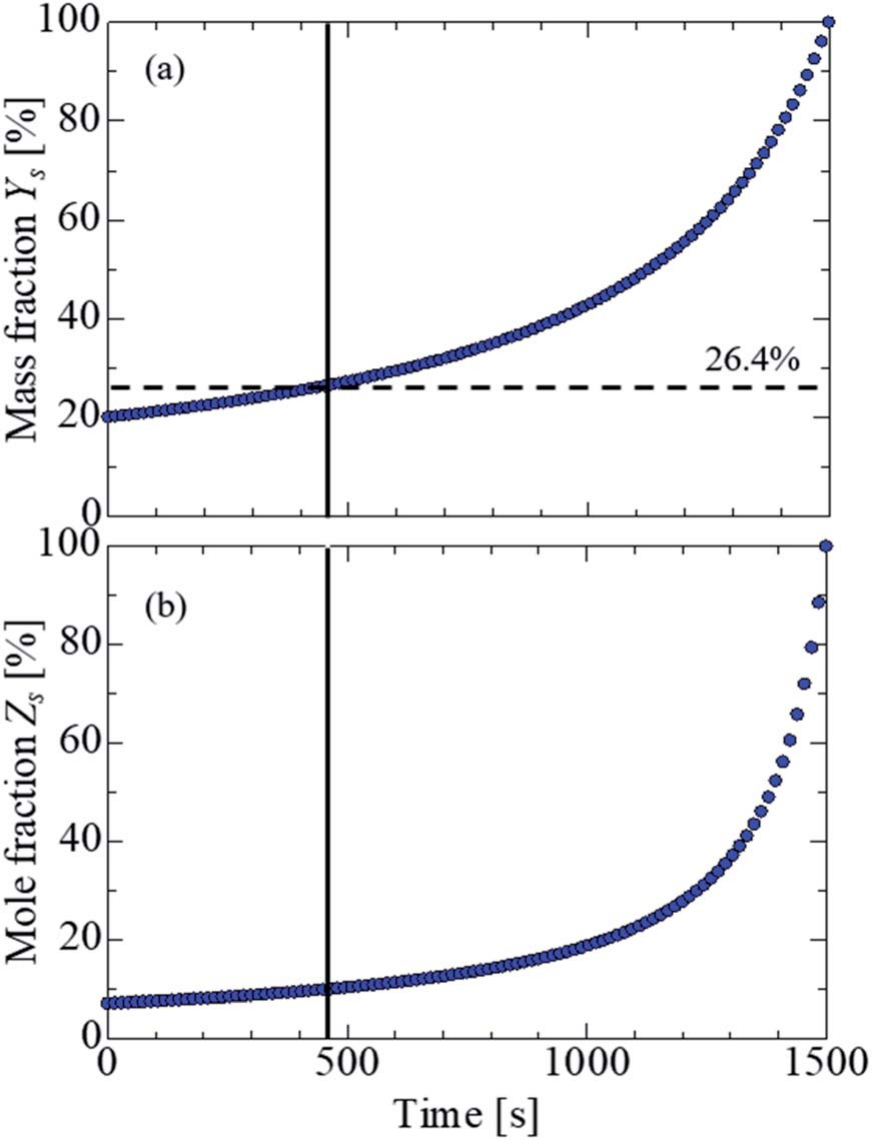
Concentration estimation at each time. (a) Mass fraction. (b) Mole fraction. The salt in water was saturated at 26.4% (dashed line) at approximately 450 s (solid line) in the present conditions.

Furthermore, to apply the obtained mass fraction at each time to the theory, the molar fraction of the solute was calculated using eqn (6).6
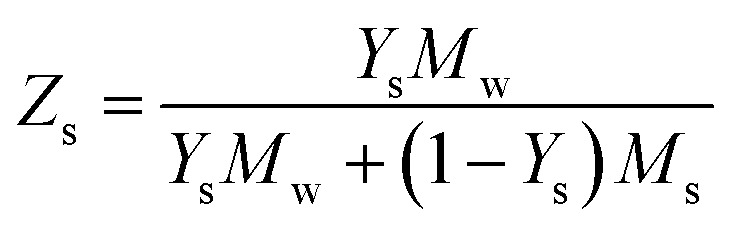
where *M* is the molar mass. Fig. 6 shows the estimation of the salinity at each time. Fig. 6(a) and (b) show the temporal evolutions of the mass and mole fraction, respectively. Because the solubility of sodium chloride in water was approximately 26.4 wt%, it was estimated that the water was saturated at approximately 450 s, and the water was completely evaporated by 1500 s. Based on this experimental data, the time evolution of the mass and mole fractions up to 450 s (saturation of sodium chloride in water) was estimated in the present study. To obtain a clear insight into the evaporation kinetics of the levitated droplet, we calculated the evaporation process considering the vapor pressure depression by using eqn (2). The red dashed line in Fig. 5 shows the theoretical value calculated using eqn (2). Although the theoretical was in partial agreement with the experimental value up to approximately 510 s, the two values exhibited considerable differences thereafter. A probable reason could be the presence of the precipitated salt on the droplet surface after the supersaturation of the salt solution. In the present study, we compared the experimental results of the salt solution droplets with the theoretical values obtained assuming an NaCl aqueous solution. Sodium chloride, however, contains electrolytes and impurities other than NaCl, and their influence must be considered in a future study.

### Time estimation for complete salt precipitation

The prediction of the evaporation and precipitation time of the levitated sample is of considerable importance for the application of the ALM in the field of chemical engineering. Thus, the precipitation time was estimated using the *d*^2^-law, considering the solute mole fraction. In eqn (2), when the droplet diameter is *d* = 0 mm, the water component of the droplet completely evaporates. Therefore, the precipitation time (complete evaporation of water) *t* can be estimated using *d* = 0 mm. Eqn (7) is used to determine the theoretical evaporation constant *β*, obtained by eqn (2).7
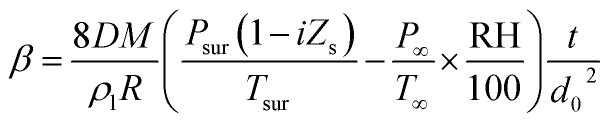


Substituting eqn (7) and *d* = 0 mm into eqn (2) and solving for *t* leads to the precipitation time *t*_p_, as shown in eqn (8).8
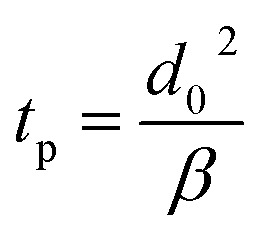


**Fig. 7:**
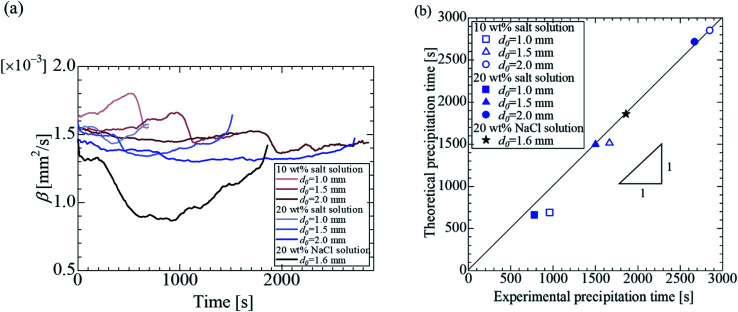
Evaporation constant *β* and time estimation for salt complete precipitation with different concentrations and diameter for different samples. (a) Evaporation constant *β* with time. (b) Comparison of experimental and theoretical precipitation times. The solid line assumes that the experimental precipitation time equals the theoretical one.

To quantify the precipitation time with eqn (8), it is necessary to calculate the evaporation constant *β*. Fig. 7(a) shows the evaporation constant up to the precipitation time (complete evaporation of water) for each sample. The horizontal axis represents time, and the vertical axis represents the evaporation constant with the different initial droplet size and concentration obtained from eqn (7). For the effect of the initial droplet diameter of the salt solution, it was confirmed that the evaporation constant decreased as the initial droplet size increased for each concentration. For the effect of the initial concentration, the evaporation constant decreased at 20 wt% for the same initial droplet diameter. Fig. 3 and 4 experimentally show that the evaporation rate decreased as the droplet size and initial concentration increased. From Fig. 7(a), these results can be explained by the change of the evaporation constant.

Based on Fig. 7 (a) and (b) shows a comparison of the precipitation time estimated using the theory of droplets with precipitation and the experimentally obtained precipitation time. The horizontal axis represents the experimental values, and the vertical axis represents the theoretically estimated precipitation time. The experimental value was calculated considered the instant at which the salt was completely precipitated and the volume equivalent diameter did not decrease during the evaporation process. The theoretical predictions were in good agreement with the experimental results. It could be concluded that the precipitation time, when the water component completely evaporates, can be predicted using eqn (7) and (8).

In this study, the effect of the vapor pressure depression was considered on the basis of Raoult's law, when determining the theoretical values. In future work, it is necessary to consider the water activity coefficient^43^ and other parameters, such as the diffusion coefficient for multicomponent droplets, a salt concentration gradient and crystallization kinetics. The equilibrium pressure of water vapor will be lower due to the concentration distribution of the salt near the interface by the evaporation of the salt droplet. This effect strongly influences the evaporation behavior, so that we demonstrated the effect of the vapor pressure depression of the droplet by eqn (2). However, the results from eqn (2) represented the effect of the average vapor pressure depression in the droplet. In our future work, we believe that it is of paramount importance to experimentally and numerically quantify the concentration distribution of salt in the droplet and discuss the evaporation and drying kinetics in more detail. For crystallization kinetics, it was a rough prediction but in good agreement with the experimental precipitation time in Fig. 7(b), even without consideration of the proper crystallization kinetics. Modelling the crystallization in the levitated droplet and the interaction with the internal flow field and precipitation is a future challenge. Another vital factor is the acoustic streaming around the levitated sample.^22–24^ Although our results demonstrated an effective prediction of the droplet evaporation and precipitation in air, the effect of the change in the thermophysical properties under the levitation and the acoustic streaming around the sample is beyond the scope of the present study.

## Conclusions

In this study, the evaporation and precipitation kinetics of multicomponent droplets *via* acoustic levitation were experimentally evaluated and compared with the expanded theoretical prediction. The salt solution droplets exhibit a two-stage evaporation process, involving water evaporation and salt precipitation. To better understand the evaporation kinetics, we presented the effect of the initial concentration and initial droplet diameter on the evaporation process. A higher concentration and larger diameter led to a lower droplet evaporation rate. Because of the vapor pressure depression, the experimental data disagreed with the classical theoretical prediction obtained using the *d*^2^-law. The experimental results and those obtained using the *d*^2^-law exhibited partial agreement when the vapor pressure depression with the concentration estimation at each time was considered using the experimental data. In addition, the precipitation time, when the water was completely evaporated, was well-predicted using the extended theory. These findings can help stimulate further research and be useful for potential lab-in-a-drop applications,^47^ such as for X-ray crystallography,^48^ blood analysis,^49^ drug development,^50^ and space experiments.^51^ Understanding the droplet dynamics during acoustic manipulation can help provide a better knowledge base for developing practical applications.

## Conflicts of interest

There are no conflicts to declare.

## Supplementary Material
